# The Fluorescent Enzyme Cascade Detects Low Abundance Protein Modifications Suitable for the Assembly of Functionally Annotated Modificatome Databases

**DOI:** 10.1002/cbic.202200399

**Published:** 2022-08-23

**Authors:** Isabel J. Hoppe, Bernhard Prommegger, Andreas Uhl, Urs Lohrig, Christian G. Huber, Hans Brandstetter

**Affiliations:** ^1^ Department of Biosciences and Medical Biology and Christian Doppler Laboratory for Innovative Tools for the Characterization of Biosimilars University of Salzburg Hellbrunner Str. 34 A-5020 Salzburg Austria; ^2^ Department of Artificial Intelligence and Human Interfaces University of Salzburg Jakob Haringer Str. 2 A-5020 Salzburg Austria; ^3^ Technical Development Biosimilars Global Drug Development Novartis Sandoz GmbH Biochemiestr. 10 A-6250 Kundl Austria

**Keywords:** analytical methods, conformation analysis, enzymes, functional annotation, protein modifications

## Abstract

Pathophysiological functions of proteins critically depend on both their chemical composition, including post‐translational modifications, and their three‐dimensional structure, commonly referred to as structure‐activity relationship. Current analytical methods, like capillary electrophoresis or mass spectrometry, suffer from limitations, such as the detection of unexpected modifications at low abundance and their insensitivity to conformational changes. Building on previous enzyme‐based analytical methods, we here introduce a fluorescence‐based enzyme cascade (fEC), which can detect diverse chemical and conformational variations in protein samples and assemble them into digital databases. Together with complementary analytical methods an automated fEC analysis established unique modification‐function relationships, which can be expanded to a proteome‐wide scale, i. e. a functionally annotated modificatome. The fEC offers diverse applications, including hypersensitive biomarker detection in complex samples.

## Graphical abstract

**Deciphering the ‘modificatome’**: We introduce fluorescent enzyme cascades which sensitively detect protein modifications and allow their functional assignment using digital pattern libraries.

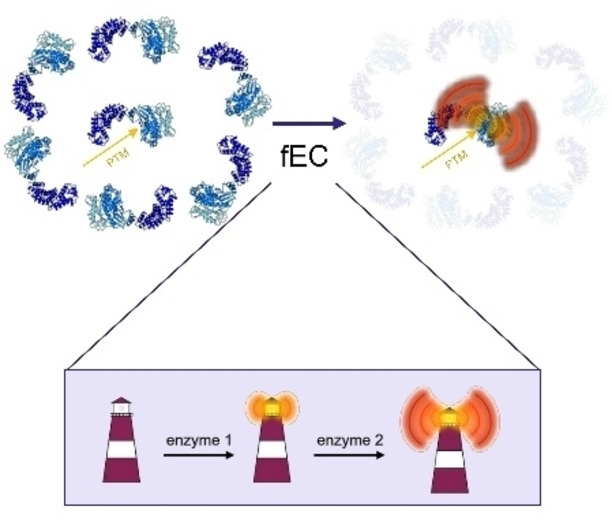

## Introduction

Proteins are universal, yet complex components of all biological systems. Their function not only depends on the primary amino acid sequence, but is critically influenced by their three‐dimensional structure.^[1]^ Correspondingly, the quality control of protein production plays an eminent role in physiology and biotechnology. In nature, complex systems of proof‐reading and elimination of misfolded or otherwise damaged proteins have been established, including heat shock proteins and lysosomal or proteasomal degradation.[^2^, ^3^, ^4^] Similarly, quality control is of critical relevance in biotechnological settings and is a pre‐requisite for reliable and reproducible production of proteins in diverse areas like research, bioengineering and biopharmaceutical industry.^[5]^ Recent studies unveiled that a majority of prominently published biomedical research could not be reproduced.[^6^, ^7^, ^8^] While there are several possible shortcomings in the processes of data production and data analysis, one important aspect is the control of protein components in the relevant experiments, i. e. educts, intermediates and products.

Current methods of quality assessment are high‐performance liquid chromatography, mass spectrometry, different spectroscopic techniques like CD, FTIR, NMR, and diverse functional assays.[^9^, ^10^, ^11^, ^12^, ^13^, ^14^] All these methods rely on bulk properties and consequently fail to detect unexpected subpopulations in the low percent range.^[9]^ For example, enzymatic activity is often considered a gold standard for correct folding of a protease; however, detailed analysis like active site titration experiments reveal that the active population is often a minority of the total protein.^[15]^ However, even minority fractions of proteins with an unwanted gain of function can cause severe side effects, for instance an unwanted immune response.[^16^, ^17^]

**Figure 1:**
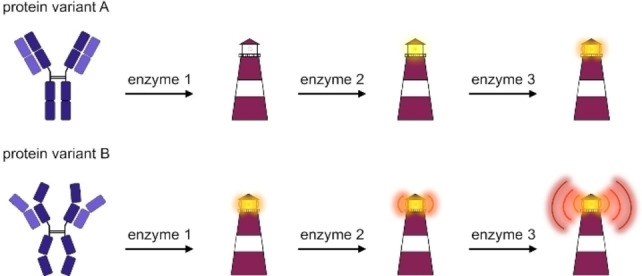
Overview of the classic enzyme cascade (cEC). Schematic representation of the method principle where subsequent enzymatic steps exponentially enhance differences between protein variants.

We recently presented a novel method to address these issues, the analytical cascade of enzymes.^[18]^ The initial, chemiluminescent version of the enzyme cascade (referred to as ACE in the original publication and cEC from here on) allows to detect small variations between protein samples, such as folding variations. To this end, the protein samples of interest are treated with a series of enzymes in order to introduce analytical modifications that exponentially enhance structural variations reflecting heterogeneities in protein samples (cf. Figure 1).

By the cascade‐based enhancement, the cEC allows to detect unknown and unexpected variations, such as post‐translational modifications, at low concentration within a given sample. Important applications are conformational changes of therapeutic or environmental proteins upon binding to nanoparticles. In a recent example, this high sensitivity could be demonstrated for the conformational changes of an allergen induced by its interaction with nanoparticles.^[19]^ Depending on details of the nanoparticle structure, only a small subpopulation of the relevant protein is affected, hence invisible to spectroscopic methods. By contrast, the cEC analysis can clearly reveal the conformational heterogeneity induced by the nanoparticle interaction.^[19]^ The positive detection of the presence of variations could in a subsequent step allow a further characterization by classical means such as mass spectrometry if needed.

With the increasing demand for cEC applications, some limitations became visible. These are mostly related to the detection step, which in the cEC implementation involves blotting onto a membrane and chemiluminescence detection. These steps are time‐intensive and inherently variable in a quantitative sense, limiting the comparability of different experiments.^[20]^ The comparison of different cEC experiments is limited by, and strictly dependent on, the availability of physical reference samples that must be analysed with every cEC experiment. Finally, the detailed quantitative analysis of cEC data is demanding and relies on a spreadsheet‐based analysis, which again is time consuming and error prone.

To address these limitations, we adapted the cEC by implementing a fluorescence‐based detection scheme, hereafter referred to as fEC. The fEC accelerates detection, and allows for absolute quantification and, in consequence, the establishment of a digital reference library. Additionally, we developed a Matlab‐based automatic data analysis routine, which should make the fEC implementation broadly accessible to academic and industry laboratories.

## Results and Discussion

### Establishing the fEC

**Figure 2:**
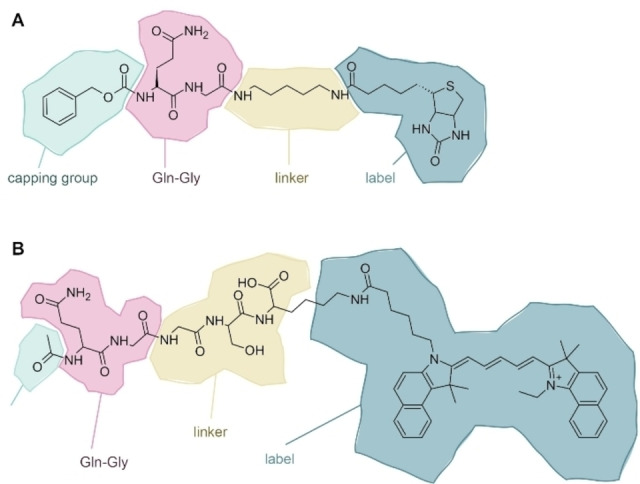
Elements required for glutamine donor peptides used in the cEC and in the first iteration of the fluorescent enzyme cascade (fEC). (A) Z−Gln−Gly−CAD‐Biotin (Gln‐biotin) used in the cEC. (B) Ac−Gln−Gly−Gly−Ser−Lys(Cy5.5)−OH used in the first fEC trials. Common elements, from left to right, are the capping group for protection of the N‐terminus (green), Gln−Gly dipeptide motif that is recognized by microbial transglutaminase (MTG) (red), linker to avoid steric problems during MTG binding and biotin‐streptavidin interaction (yellow), and detection label (blue). Both structures were drawn using ACD/ChemSketch 2020 1.2 (ACD/Labs, Toronto, Canada).

The initial cEC consists of several analytical enzymatic modifications which enhance small changes in protein samples. The final modification is a transglutamination reaction which enables a readout of the EC modification. Hereby, we use a microbial transglutaminase (MTG), which covalently links lysine and glutamine side chains, resulting in isopeptide bond.^[21]^ In previous studies we found it favourable for modification yield to attach a Gln‐based peptide to protein‐based lysine residues.^[18]^ The schematic composition of a Gln‐based ligand is shown in Figure 2A. It consists of (i) an N‐terminal capping group (green), (ii) a Gln‐Gly dipeptide (red) reflecting the substrate specificity of MTG,[^21^, ^22^] (iii) a linker / spacer (yellow) minimizing the interference with the transglutamination reaction, and (iv) a label such as biotin (blue) which can be employed to visualize the modification reaction. The ligand shown in Figure 2A fulfilled all requirements.^[18]^ To enable direct visualisation and absolute quantification, we designed a first successor ligand with a Cy5.5 fluorophore replacing the biotin label (Figure 2B).

**Figure 3:**
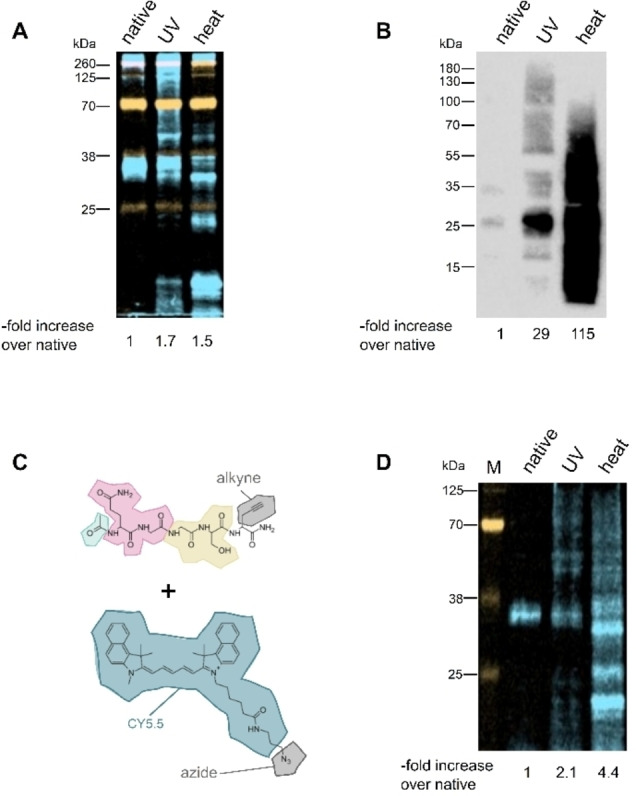
Comparison of initial fEC results with the cEC. Lane 1: native Rituximab, lane 2: UV‐stressed Rituximab, lane 3: heat‐stressed Rituximab. Overall signal intensity increase over the native sample is indicated under each lane (A) First generation of the fEC: Transglutamination carried out using Gln‐CY5.5 label, samples resolved on SDS‐PAGE and imaged directly in gel cassette. Orange: 800 nm (MW ladder), blue: 700 nm. (B) cEC: Transglutamination carried out using Gln‐biotin label, samples resolved on SDS‐PAGE, blotted to membrane, and detected via chemiluminescence. (C) Ligand and fluorescent label used for the second generation fEC, colour code identical to Figure 2, alkyne‐azide pair for CuAAC in grey (D) Second generation of fEC: Transglutamination carried out using Gln‐alkyne, modified protein labelled with CY5.5 via copper‐catalysed azide‐alkyne cycloaddition (CuAAC),^[23]^ samples resolved on SDS‐PAGE and imaged directly in gel cassette. Marker M in orange: 800 nm, blue: 700 nm.

To evaluate the effect of this modified ligand, we used a simple two‐step EC consisting of legumain cleavage followed by MTG transglutamination and a well characterized target protein, the antibody Rituximab, under different stress conditions. Remarkably, the degree of labelling in the stressed protein variants (UV‐light, heat treatment) was only slightly elevated as compared to the native protein (Figure 3A). These 1.7 and 1.5‐fold signals were in stark contrast to the enhancement effect seen in the classical EC detection protocol with 29‐ and 115‐fold signal enhancement, respectively (Figure 3B). This suggests that the modification of the ligand, as shown in Figure 2, affected and drastically deteriorated the sensitivity of the EC detection in this first generation fEC. The low sensitivity prompted us to systematically test the effects of the different components of the ligand.

Given that the N‐terminal Gln‐Gly dipeptide is strictly required by the transglutaminase as a recognition motif, it must remain conserved. Due to its steric and hydrophobic properties, we expected the fluorophore to have a pronounced effect on the EC reaction. We therefore started by evaluating the effect of the newly introduced fluorophore group on the MTG reaction. To this end we replaced the fluorophore by an alkyne group (Figure 3C), which allowed us to uncouple the transglutamination and the labelling steps by bio‐orthogonal click chemistry with azide‐based labels. Avoiding the fluorophore in the transglutamination reaction indeed improved the fEC signal by approximately two‐fold (Figure 3D), but it was still far less sensitive than the biotin‐labelled cEC.

**Figure 4:**
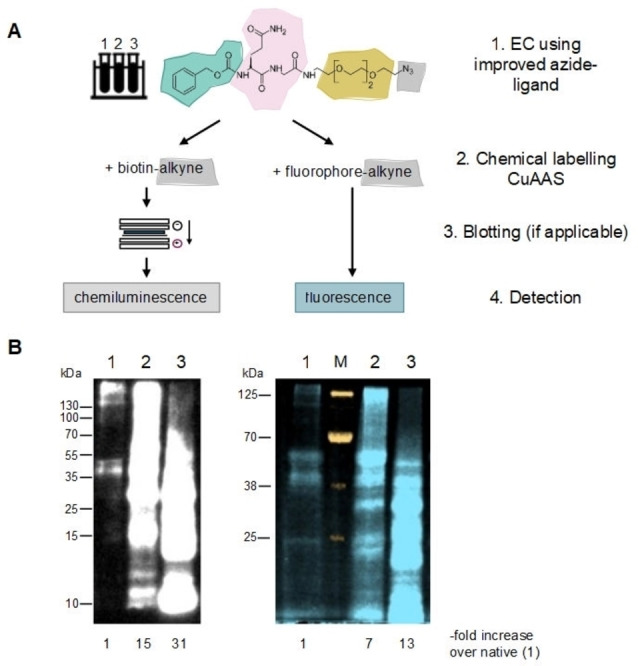
Evaluation of the effect of ligand composition, blotting process and detection method on the differential signal achieved in the EC. (A) Schematic of the experimental workflow. 1. EC treatment of three different Rituximab samples (1: native, 2: UV‐stressed, 3: heat‐stressed) using an improved mTG ligand containing an azide for CuAAS; 2. All samples divided in two and labelled via CuAAC with a biotin‐ and a fluorophore‐alkyne; 3. All samples were resolved on SDS‐PAGE, biotin‐labelled samples were then blotted onto a membrane and detected via chemiluminescence, fluorescence‐labelled samples were imaged at 700 nm directly in the SDS‐PAGE cassette. (B) Results of the experiment, arranged under the corresponding experimental setup from (A). Lane 1: native, lane 2: UV‐stressed, lane 3: heat‐stressed. White: chemiluminescence, orange: 800 nm (MW ladder M), blue: 700 nm. Overall signal intensity increase over the corresponding native sample is indicated under each lane.

The comparison of the ligands in the cEC (Figure 2A) and in this second generation fEC (Figure 3C) pointed towards the linker within the ligand as a critical determinant of EC sensitivity. To address this possibility, we designed a next ligand which replaced the peptide‐based linker by a PEG_3_‐linker. PEGylation has proven to be a particularly biocompatible chemistry and is used for the modification of biotherapeutics, e. g. to prolong the half‐life in serum.^[24]^ We performed the click‐mediated labelling with both a fluorophore and a biotin as a control. While the biotin‐labelled sample required an additional blotting step before detection, the fluorophore‐labelled sample could be detected directly in the gel (Figure 4A). The EC reaction with the new ligand containing a PEG linker enhanced the sensitivity dramatically, qualitatively restoring the signal as known from the cEC (Figure 4b). In the scientific literature the observed interference of poly‐glycine/serine‐linkers may be underappreciated, given their common usage as linkers in protein engineering, for example for the design of fusion constructs.^[25]^ The alkyne‐azide click reaction further enabled us to assess the potential effect exerted by the blotting step necessary for detection in the cEC, which is another difference in cEC and fEC. By also blotting the fluorophore‐labelled sample before detection, we could show that the blotting step had no further effect on the EC sensitivity (Figure S1).

### Broad applicability of fEC

**Figure 5:**
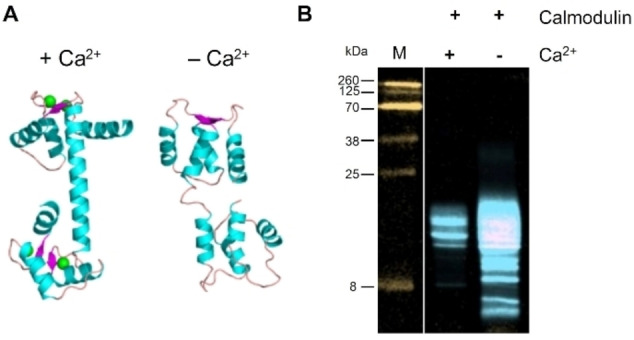
Application example of the fEC. (A) Structural changes in calmodulin. Ca^2+^‐bound calmodulin (PDB: 3CLN) vs. apo‐calmodulin (PDB: 1CFC). Green: Ca^2+^, cyan: α‐helix, pink: β‐sheet, peach: coil. (B) Detection of structural changes in calmodulin caused by depletion of calcium (achieved by addition of EDTA) by two‐step fEC. Orange: 800 nm (MW ladder M), blue: 700 nm.

Initial EC studies were triggered by the interest to assess the quality of therapeutic proteins. Therefore, we investigated different antibodies such as Rituximab.^[18]^ Further, we investigated whether the cEC analysis can detect purely conformational or covalent modifications in proteins that are not as robust as therapeutic antibodies. Here, we used the well characterized protein calmodulin to assess strengths and possible limitations of the fEC in detecting different conformers. To induce different calmodulin conformations, we analysed the protein in the presence and the absence of calcium (Figure 5). Calmodulin is known to have four calcium binding sites and to undergo large structural changes upon calcium binding (Figure 5A).^[26]^ We performed a two‐step fEC analysis, comprising a proteolysis and a transglutamination reaction (Figure 5B). Both chymotrypsin and papain proved suitable for the proteolysis reaction, while MTG was used as a labelling enzyme. To optimise the signal to background, we reduced the protease concentration in the first EC step by more than thousand‐fold, which resulted in the desired limited proteolysis of calmodulin. The standard parameters as used for antibodies resulted in an over‐digestion of calmodulin (Figure S2).

Calcium binding is prevented at acidic pH, where carboxylate groups become protonated, unable to chelate the calcium. Consequently, we performed the fEC reaction at neutral pH. We added EDTA to extract calcium from calmodulin. To avoid interference with the subsequent CuAAC reaction, it proved vital to saturate the EDTA prior to the CuAAC reaction.

### Absolute quantification of fEC labelling

**Figure 6:**
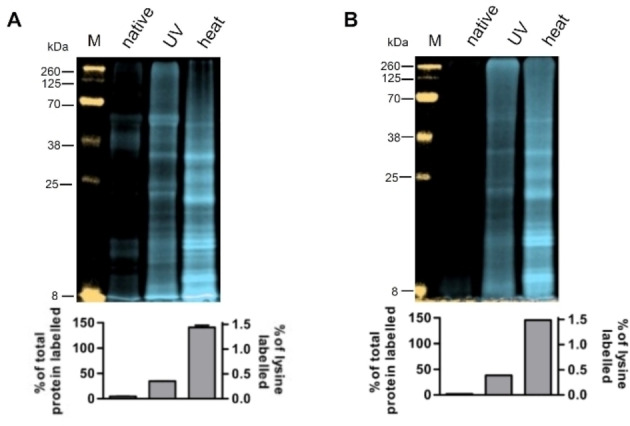
Absolute quantification and one‐pot fEC labelling. Orange: 800 nm (MW ladder M), blue: 700 nm. (A) Sequential, 2‐step fEC of three different Rituximab samples. Absolute quantification of the degree of labelling shown in the bar graph underneath each lane. At high Cy5.5 emission intensity a slight orange colour cast is visible, which is caused by the Cy5.5 emission shoulder at 800 nm. (B) One‐pot fEC of three different Rituximab samples. Proteolysis and transglutamination were carried out simultaneously instead of sequentially. Absolute quantification of the degree of labelling shown in the bar graph underneath each lane.

In contrast to the western blot‐based detection in the cEC, the fluorescence labelling in fEC offers the advantage of absolute quantification. The cEC relied on the indirect detection and signal amplification via horseradish peroxidase, making quantification of the degree of labelling and therefore comparison across multiple experiments impossible. It was possible to perform a simple dilution series of the fluorophore. Using a standard curve, we then calculated the absolute molar amount of fluorophore coupled to the target protein and from this, the percentage of total protein that had been labelled during the fEC. In the example shown here (Figure 6A), approximately 4.7 % of native Rituximab (corresponding to 0.05 % of the lysine residues contained in the protein) was labelled, while 34.7 and 141.0 % of UV‐ and heat‐stressed Rituximab (corresponding to 0.35 and 1.44 % of the lysine residues) was labelled, respectively.

For the interpretation one must consider that the labelling may not be homogeneously distributed over the protein population. Instead, a small “contaminant” fraction of the protein of interest may differ in its conformation, e. g., partial glycosylation or phosphorylation. Specifically, an apparent 10 % labelling in a protein sample can be interpreted as (i) a homogeneous sample with every 10th molecule labelled at one lysine residue, or (ii) every hundredth molecule labelled at 10 lysine residues. In fact, this property is a unique strength of the EC, which allows to sensitively detect also minor, but often highly relevant, subpopulations within a complex sample.[^16^, ^17^]

To further streamline the fEC protocol, we tested whether the enzymatic modification steps could be combined into a one‐pot reaction with a reaction condition (such as pH, temperature, salt) compatible with all used enzymatic reactions. To test the applicability of the one‐pot ACE, we used the same Rituximab variants as for the sequential fEC. As shown in Figure 6B, this simplified ACE analysis was still able to clearly distinguish between the different modifications. Furthermore, quantification of the degree of labelling gave very similar values to those seen in the sequential fEC (2.1, 38.3 and 145.7 % respectively for native, UV‐ and heat‐stressed protein). The signal enhancement depends on the availability of transglutamination sites for protein labelling. The increased accessibility depends on the partial destabilization of the target protein by modifications such as cleavage or citrullination, but apparently not necessarily on the sequence of these modifications. The detailed labelling pattern may, however, vary between a sequential fEC and a one‐pot fEC and they should not be used interchangeably.

### Automated analysis of fluorescent SDS‐PAGEs

**Figure 7:**
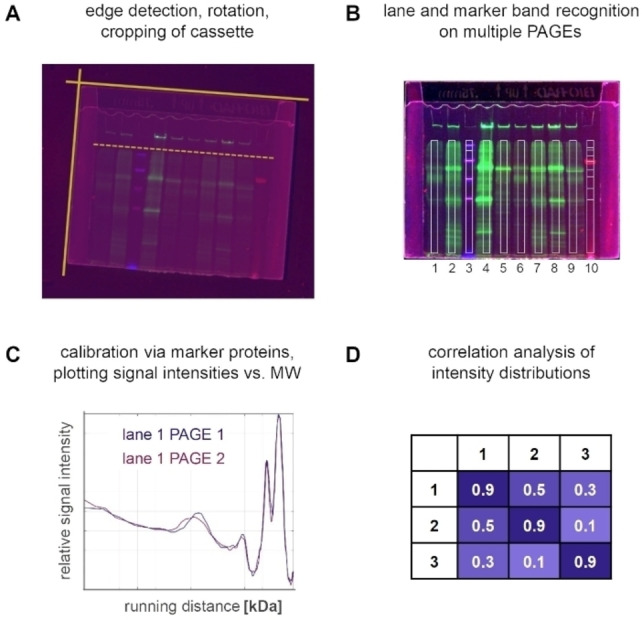
Overview of the automated analysis of fEC data. (A) Gel cassette recognition. The software recognizes characteristic edges, including the gel boundaries and the border between stacking and resolving gel (dashed line). Subsequently the gel was rotated to optimize the vertical alignment of the gel lanes, Figure 7A. (B) Marker band assignment. In a next step individual lanes were assigned with the bottom of the gel cassette and the top of the resolving gel serving as the lanes’ lower and upper boundary, respectively. The width of the lanes was calculated from the specified number of lanes per gel. The fluorescently labelled marker bands were recognized by their prominence and assigned as per specification, Figure 7B. (C) Calibration of sample bands. For the mass assignment, the information of the defined marker proteins is used, allowing for a mass assignment to each running distance in the SDS‐PAGE by inter‐ and extrapolation. The fluorescent intensities representing the protein bands are then plotted against the molecular weight for each lane (Figure 7C). (D) Correlation analysis. With consistent mass assignment, fEC fluorescence intensities can be correlated and compared quantitatively across different experiments (Figure 7D).

Our current findings suggest that the EC analysis will work with the large majority of proteins, with minor modifications of the EC conditions specific to the target protein.[^18^, ^19^] As illustrated by the calmodulin example (Figure 6), the fEC analysis clearly revealed the difference between the two calmodulin proteoforms, however, the analysis of the fEC chromatograms alone do not reveal the molecular basis of these differences. Such mechanistic details can be obtained from fEC data by correlation analysis with fEC reference libraries of well‐defined or characterised proteoforms, as described below (Figure 7).

One of the major advantages of fEC's direct fluorescent signal readout as compared to the cEC is the possibility to compare fEC patterns over different gels, allowing to assemble electronic fEC libraries for rapid protein analytics. To compare fEC profiles on different gels, the fluorescence intensities in each lane must be assigned to molecular masses that serve as reference for all lanes on different gels. This step is critical because of the inherent variability of SDS PAGEs due to different parameters (voltage, percentage and heterogeneity of the gel and running time, among others).

However, with these possibilities the demand for automated analysis of digitalised PAGEs arises, which should increase the throughput and render the analysis less prone to errors. To this end, we developed a Matlab‐based program. The code is available as a supplement to this publication.^[27]^ The automated analysis comprises the following four steps, shown in Figure 7.

### Classification of structural impurities in Rituximab using a digital reference library

One of the attractive features of the cEC was its use in the characterization and assignment of diverse modifications of a protein of interest. This builds upon the observation that different modifications are reflected by distinct intensity and mass patterns in the EC analysis. By performing the EC on reference stress conditions and comparing the patterns of these to unknown samples via correlation calculation, it is possible to determine the type of modification.^[18]^ We carried out different treatments on the test protein (native, UV irradiation, deglycosylation of Rituximab) and employed the fEC analysis of the resulting Rituximab variants. Correlation analysis revealed an unambiguous assignment of the different modification types (Figure S3). Given the inherent quantification by the direct fluorescence detection, the fEC analysis offers an important advantage over the cEC analysis. While in the cEC it was necessary to provide both the reference modifications and the test modifications on the same SDS‐PAGE, it should be possible to assemble an electronic reference database of fEC fluorescence patterns matching defined protein modifications. In an independent fEC experiment it should then be possible to assess the (unknown) modification of the protein of interest by a correlation analysis of its fEC profile with the electronic reference database.

**Figure 8:**
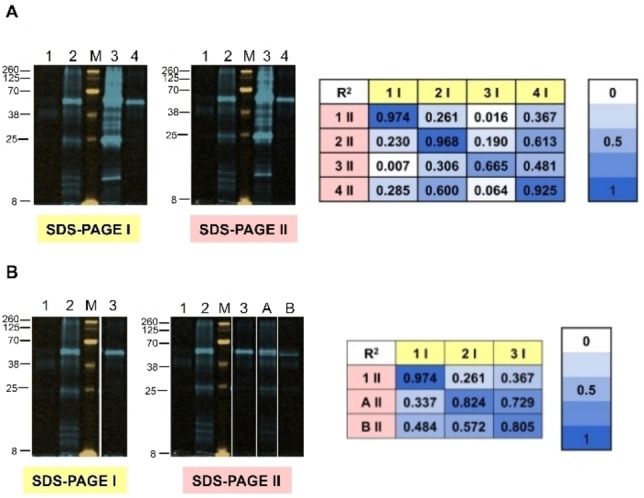
Classification of structural impurities in Rituximab. Orange: 800 nm (MW ladder M), blue: 700 nm. (A) Library samples and identical replicates thereof (1. native, 2: UV‐stressed, 3: heat‐stressed, 4: deglycosylated protein) after two‐step fEC (limited proteolysis using pepsin followed by transglutamination) run on two different SDS‐PAGE gels to show reproducibility between gels. Heat map shows the squared correlation coefficient (R^2^) determined by the described MatLab script. (B) Library samples (1: native, 2: 10’ UV stress, 3: full deglycosylation) and variations thereof (A: 5’ UV stress, B: 20 % deglycosylated protein spiked into native sample) after two‐step fEC (limited proteolysis using pepsin followed by transglutamination), analysed on two SDS‐PAGE runs. Heat map shows the squared correlation coefficient (R^2^) determined by the described MatLab script.

To determine whether digital reference libraries can indeed be used in the fEC analysis, we initially tested whether it would be possible to accurately correlate identical fEC samples across two independent SDS‐PAGE gels (Figure 8A). We resolved the same samples of native, UV‐stressed, heat‐stressed, and deglycosylated Rituximab on two separate SDS‐PAGE gels. The corresponding fEC fluorescence gels were then digitized and correlated by our Matlab‐based prototype (see Figure 7). The resulting heatmap showed that the samples can be correctly assigned across independent SDS‐PAGE runs.

Next, we tested whether non‐identical modifications could be correctly assigned. Accordingly, a reference library was generated consisting of three different known Rituximab variants. On a separate gel, test samples of Rituximab were analysed, which differed in detail from the defined reference conditions (Figure 8B). Specifically, the reference conditions comprised Rituximab in its native state, after UV exposure for 10 min, and after deglycosylation by PNGase, overnight. The test samples consisted of Rituximab after UV exposure for 5 min and after simulated partial deglycosylation implemented by dilution of 20 % deglycosylated protein into native protein. The resulting fluorescence gels were digitized and correlated by our Matlab‐based prototype. We found an unambiguous correlation assigning each test condition to the closest matching reference sample, thereby demonstrating the transferability of the fEC profiles over different experiments.

## Conclusion

The fluorescent enzyme cascade furnished several important advantages over the chemiluminescent enzyme cascade while preserving its strengths, in particular the detection of low abundance modifications in complex samples with unmatched sensitivity. In its one‐pot format the fEC assay can be carried out in half a day and yields absolute quantification of its results.

The flexible nature of the azide‐based labelling in the fEC opens the door for further development options, such as the transfer to HPLC‐based separation and fluorescence detection.

With the use of multi‐dimensional digital reference libraries, the qualitative type of modification and its quantitative extent can be assessed. Of high practical future relevance, fEC results can be collected and made available at web repositories, to assemble a growing encyclopaedia of protein modifications with physiological, pathological, or environmental functions assigned to them. The direct fEC analysis of complex samples offers the potential to detect transient, matrix‐dependent proteoforms, and will require the assembly of high‐quality reference libraries. Alternatively, a specific separation step, e. g. immunoprecipitation, can be performed either preceding or subsequent to the enzyme cascade treatment.

Such massive libraries of proteome‐wide modifications (the modificatome) critically depend on an automated data analysis as implemented by our Matlab‐based prototype. In fact, the fEC also meets the three key criteria for machine learning analysis, i. e. (i) massive combinatorial search space, (ii) clear objective metric to optimise against, and (iii) lots of data.^[28]^ Together, these features make the fEC an attractive method for many relevant applications, including hypersensitive biomarker detection in disease areas like cancer or neurodegeneration, where early detection is critical.

## Experimental Section

All details can be found in the Supporting Information.

## Conflict of interest

Hans Brandstetter holds a patent on the enzyme cascade based detection method (US patent 10,775,384).

1

## Supporting information

As a service to our authors and readers, this journal provides supporting information supplied by the authors. Such materials are peer reviewed and may be re‐organized for online delivery, but are not copy‐edited or typeset. Technical support issues arising from supporting information (other than missing files) should be addressed to the authors.

Supporting Information

## Data Availability

The data that support the findings of this study are available from the corresponding author upon reasonable request.
