# Supplementary material for: The Fluorescent Enzyme Cascade Detects Low Abundance Protein Modifications Suitable for the Assembly of Functionally Annotated Modificatome Databases
Source: Chembiochem. 2022 Aug 23;23(19):e202200399. doi: 10.1002/cbic.202200399 (PMC9805100; doi:10.1002/cbic.202200399)
Supplement: Supplementary file 1 — Supporting Information [file CBIC-23-0-s001.pdf]

ChemBioChem

Supporting Information

The Fluorescent Enzyme Cascade Detects Low Abundance Protein Modifications Suitable for the Assembly of Functionally Annotated Modificatome Databases

Isabel J. Hoppe, Bernhard Prommegger, Andreas Uhl, Urs Lohrig, Christian G. Huber, and Hans Brandstetter*

Table of Contents

Experimental Procedures 1
Materials 1
Rituximab derivatisation 2
Quantification of fluorophore labelling 2
Enzyme cascade protocols 3
Matlab prototype for classification of structural variations 4
Results and Discussion 5
References 6
Author Contributions 6

Experimental Procedures

Materials

Rituximab (MabThera) was purchased from Hoffmann-La Roche. Activated human wild type legumain was purchased from Jena Bioscience or produced in-house as described previously [1]; PNGase F from *Elizabethkingia meningoseptica* was recombinantly expressed in-house as described previously [2] using a plasmid provided by Manuel Than, FLI Jena or, alternatively, purchased from New England Biolabs; papain from *Carica papaya*, pepsin from porcine gastric mucosa and calmodulin from bovine brain were purchased from Merck (Darmstadt, Germany), and recombinant microbial transglutaminase (MTG) from *Streptomyces mobaraensis* was purchased from Zedira. Transglutaminase substrates and click chemistry labels were purchased from suppliers as listed in Table S1 and S2, respectively. Cy5.5 was chosen based on the available detection instrument (Licor Odyssey Fc), which offers detection at 700 and 800 nm. Due to the uncoupling of the (chemical) labelling reaction from the (enzymatic) signal enhancement many other wavelengths or labelling techniques should be possible. All other chemicals used were purchased from Merck (Darmstadt, Germany), unless stated otherwise.

Table S1. Glutamine-donor peptides.

Name **(abbreviation)**	Structure	Source
Z-Gln-Gly-CAD-Biotin **(Gln-biotin)**	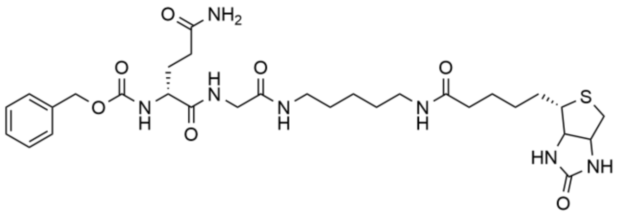	Zedira GmbH, Darmstadt, Germany
Ac-Gln-Gly-Gly-Ser-Lys(Cy5.5)-OH **(Gln-Cy5.5)**	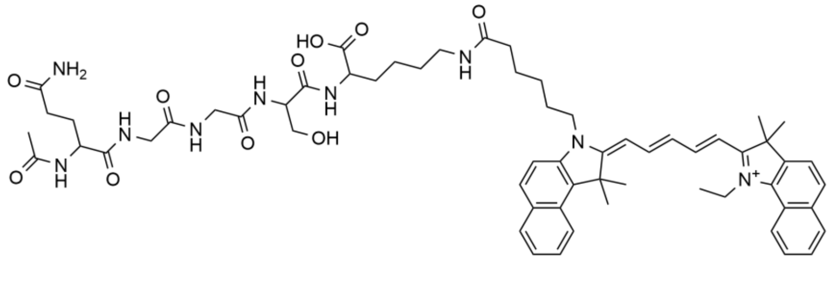	Pepscan, Lelystad, The Netherlands custom synthesis

Ac-Gln-Gly-Gly-Ser-Pra Pra=propylargyl-L-glycine **(Gln-alkyne)**	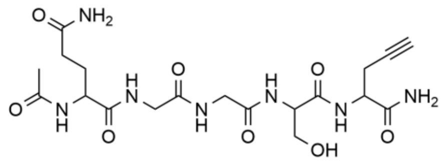	JPT Peptide Technologies, Berlin, Germany custom synthesis
Z-Gln-Gly-PEG$_3$-N3 **(Gln-azide)**	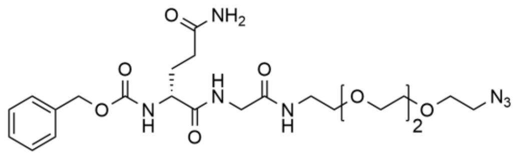	Zedira GmbH, Darmstadt, Germany

Table S2. Click-chemistry labels.

Name **(abbreviation)**	Structure	Source
Cyanine5.5 azide **(Cy5.5-azide)**	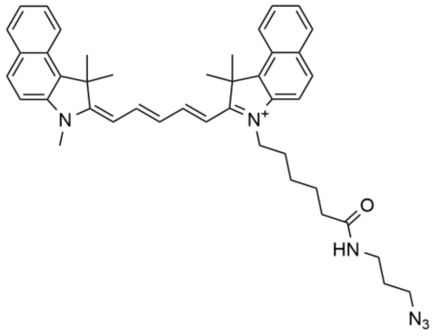	Lumiprobe, Hannover, Germany
Biotin-PEG$_4$-alkyne **(Biotin-alkyne)**	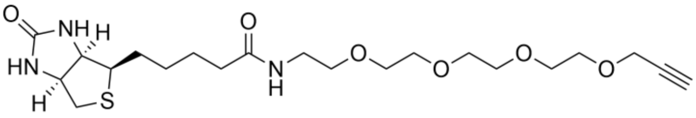	Sigma-Aldrich, St. Louis, USA
Cyanine5.5 alkyne **(Cy5.5-alkyne)**	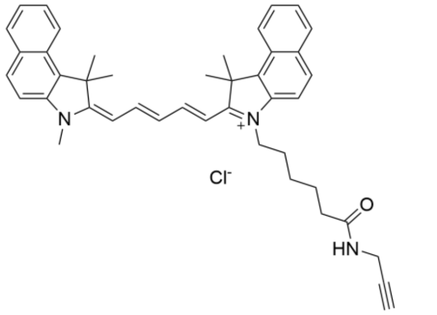	Lumiprobe, Hannover, Germany

Rituximab derivatisation

All buffer exchange steps were carried out using Illustra NAP-5 columns (GE Healthcare) following the manufacturer's instructions. For native, UV- and heat-stressed conditions, Rituximab was rebuffered into 20 mM sodium acetate, 20 mM NaCl, pH 4.5, except for experiments in Figs. 3a and b, where it was rebuffered into 100 mM sodium acetate, 20 mM NaCl, pH 4.5. Samples were then diluted to the target concentration in the same buffer. For native samples, Rituximab was used without further treatment. For UV-stressed samples the tube was placed on top of a 2UV Transilluminator (UVP) for 5 or 10 min, as indicated. For heat-stressed samples, the protein was incubated at 80 °C for 5 min, followed by immediate cooling on ice.
For deglycosylation, Rituximab was re-buffered into 50 mM sodium phosphate pH 7.4 and incubated with PNGase F (1:300 ratio of PNGase F:Rituximab by weight) at 37 °C for 18 h, then buffer exchanged into 20 mM sodium acetate, 20 mM NaCl.

Quantification of fluorophore labelling

To enable quantification of the degree of labelling in fEC samples, a standard curve for the CY5.5-alkyne label was created. A two-fold dilution series ranging from 20 µM to 2.4 nM was prepared in duplicate, and a 4 µl drop of each dilution was pipetted on top of two hand-cast SDS-PAGE gels, which were then imaged with the 700-channel. The signal intensities were determined by drawing a rectangle with local background subtraction around each spot using Licor Image Studio Lite Ver 5.2, and a standard curve of the amount of substance of CY5.5-alkyne vs. signal intensity was calculated using Microsoft Excel 2016. To determine the degree of labelling of a given sample, a rectangle was drawn around the corresponding lane on the SDS-PAGE to measure total signal intensity, and the amount of CY5.5 labels was calculated from the standard curve.

Enzyme cascade protocols

All incubation steps were carried out at 37 °C. All ratios given are molar ratios. All samples were prepared for SDS-PAGE by addition of 1:4 volume of 0.2 M Tris-HCl, 8% SDS, 40% glycerol, 0.16% Orange G, 0.4 M DTT, pH 6.8, followed by denaturation at 95°C for 10 min, and 5 µg of protein per lane were resolved on handcast 14% SDS-PAGE gels unless otherwise specified. An Odyssey Fc Imaging System (LI-COR Biosciences) was used for fluorescence detection of resolved fEC samples directly in the SDS-PAGE cassette.
The cEC shown in Fig. 3b, was carried out as described previously [3] with a starting concentration of Rituximab of 2 mg/ml, 1:50 Legumain:Rituximab, 1:5 MTG:Rituximab and 300:1 Gln-biotin:Rituximab.

First generation fEC Native, UV-, and heat-stressed Rituximab at a concentration of 0.6 mg/ml was first incubated with 1:50 Legumain:Rituximab for 2h. The protease was then inhibited by addition of 10 µM Ac-YVAD-cmk (Bachem). Transglutamination was carried out using 1:5 MTG:Rituximab and 1:1 Rituximab:Gln-CY5.5 (see Tab. S1) for 3h. The reaction was stopped by addition of reducing SDS-PAGE sample buffer.

Second-generation fEC Native, UV-, and heat-stressed Rituximab at a concentration of 1 mg/ml was first incubated with 1:50 legumain:Rituximab for 2h. The protease was then inhibited by addition of 10 µM Ac-YVAD-cmk. Transglutamination was carried out using 1:5 MTG:Rituximab and 1:150 Rituximab:Gln-alkyne (see Tab. S1) for 3h. The reaction was stopped by addition of 10 mM N-ethylmaleimide. The samples were then diluted with an equal volume of 0.2M potassium phosphate pH 7.0 and the labelling was carried out via copper-catalyzed alkyne-azide cycloaddition (CuAAC) [4]. The reactions specifically contained 0.425 mM alkyne (stemming from the fEC reaction), 0.85 mM (i.e. a two-fold excess of) Cy5.5-azide (see Tab. 1), 0.1 mM $CuSO_4$, 0.5 mM Tris(3-hydroxypropyltriazolylmethyl)amine (THPTA), and 5 mM sodium ascorbate. Tubes were capped, wrapped in aluminium foil, and incubated at RT for 1h. To stop the CuAAC reaction, 5 mM EDTA was added, and the samples were prepared and visualized as described.

Third-generation fEC. For the experiment shown in Fig. 4, native, UV-, and heat-stressed Rituximab at a concentration of 0.7 mg/ml was first incubated with 1:50 legumain:Rituximab for 2h. The protease was then inhibited by addition of 10 µM YVAD-CMK. Transglutamination was carried out using 1:5 MTG:Rituximab and 150:1 Gln-azide:Rituximab (see Tab. 1) for 3h. The reaction was stopped by addition of 10 mM N-ethylmaleimide. The samples were then diluted with an equal volume of 0.2M potassium phosphate pH 7.0 and the labelling of each sample with both biotin and a fluorescence dye was carried out via CuAAC [4]. The reactions specifically contained 0.28 mM azide (stemming from the fEC reaction), 0.56 mM biotin-alkyne or Cy5.5-alkyne (see Tab. S1), 0.25 mM $CuSO_4$, 1.25 mM THPTA, and 5 mM sodium ascorbate. Tubes were capped, wrapped in aluminium foil, and incubated at RT for 1h. All samples were resolved on SDS-PAGE as described above, immunoblotted as described previously [3] and detected via either fluorescence imaging or chemiluminescence using the ECL prime western blotting detection kit (GE Healthcare) according to the manufacturer's instructions. Fluorescence-labelled samples were also imaged directly in the SDS-PAGE cassette.
For the experiments shown in Figs.6a and 8, enzyme cascades were carried out as described for Fig. 4 with minor changes. Specifically, the starting concentration of Rituximab was 0.6 mg/ml, proteolysis was carried out using legumain for Fig. 6a and pepsin (Sigma-Aldrich) for Fig. 8, both with 1.5 h incubation. Pepstatin A (Sigma-Aldrich) at 10 µM was used to inhibit pepsin. The transglutamination reaction was allowed to proceed for 2h. For fluorescent labelling, the samples were diluted with an equal volume of 200 mM sodium phosphate pH 7.0, and the concentrations of azide (stemming from the EC reaction) and CY5.5-alkyne were 0.23 and 0.46 mM, respectively. To remove excess fluorophore, acetone precipitation was carried out [5]. The one-pot fEC reactions shown in Fig. 6b were carried out identically, except that legumain, MTG, and the Gln-azide ligand were added at the same time and incubation time was 2h. No protease inhibitor was therefore necessary. In the shown implementation of the experiment, we performed an acetone precipitation prior to SDS-PAGE, allowing us to wash away the excess fluorophore (CY5.5-alkyne) from the reaction mixture and reduce the background signal accordingly. Our fluorophore exhibited a low electrophoretic mobility in the PAGE, interfering with protein sample detection. Depending on the physical properties of the used label, this step may not be necessary.
Variation for calmodulin The fEC with calmodulin was carried out as described for the third generation fEC, with minor adjustments. Calmodulin (from bovine brain, EMD Millipore) was prepared in 30 mM HEPES, 1 mM $CaCl_2$, pH 7.4, ± 5 mM EDTA at a starting concentration of 1 mg/ml. For proteolysis, 1:10.000 papain (Merck):calmodulin was used with an incubation time of 1h, followed by papain inhibition with 10 µM E-64 (Sigma-Aldrich). The transglutamination was carried out using 1:50 MTG:calmodulin and 50:1 Gln-azide for 2h. After inhibition of MTG, the EDTA in the reaction was saturated by addition of 10 mM $CaCl_2$ as a sacrificial metal. Labelling by CuAAC was carried out directly, in the same buffer as the EC, with concentrations of 1.5 mM and 3 mM for the azide and CY5.5-alkyne, respectively.

Matlab prototype for classification of structural variations

The automation of the fEC data analysis was implemented with a Matlab-based prototype. The functionality includes detection of the SDS-PAGE cassettes and lanes; automatic recognition of molecular weight marker proteins, enabling the conversion of running distances to the molecular weights of the protein bands. This absolute mass assignment allows the calculation of the correlations between unknown test samples and digital reference samples, i.e. digital libraries. The Matlab prototype carries out such correlation analyses automatically.

The prototype currently expects raw data from Licor ImageStudio and samples run on a 10-well hand-cast Mini-PROTEAN polyacrylamide gel (Bio-Rad Laboratories). ImageStudio provides separate images for each acquired near infrared channel (excitation at approximately 600, 700, and 800 nm). For further processing, the three channels are combined into a single image. The position of the cassette on the input image is determined with the help of edge detection. The recognized cassette is rotated for horizontal alignment and the image is cropped. In the next step, the lane regions are defined. The horizontal positions as well as the width of the lanes are given by the design of the cassette. The vertical start of the lane is determined by edge detection of the transition from stacking to separating gel. Fluorescence intensities are determined by averaging the signal intensity over the width of the lane.

The marker proteins are identified by detecting local fluorescence maxima in the channel matching the marker fluorophore emission. The molecular weights of the sample protein bands are calculated by interpolation of the marker protein bands using Matlab's exp2 two-term exponential regression model. The type of protein ladder and the lane(s) in which it is run can be configured. Currently there are two supported protein ladders: Chameleon 800 Pre-stained Protein Ladder (Li-Cor Biosciences) and PageRuler Prestained Protein Ladder (Thermo Fisher Scientific).

As a result, average fluorescence intensities are obtained as a function of their molecular weight for each lane. The prototype correlates different protein samples with reference libraries using Matlab's cross-correlation function. Alternatively, Pearson's linear correlation coefficient can be used. All processing steps can be manually corrected, for example manual adjustment of the lane positions in case of incorrect identification by the software. Further details on the prototype, including exhaustive instructions, reference datasets and the Matlab source code can be found on https://plus.ac.at/xray/repository.[6]

Results and Discussion

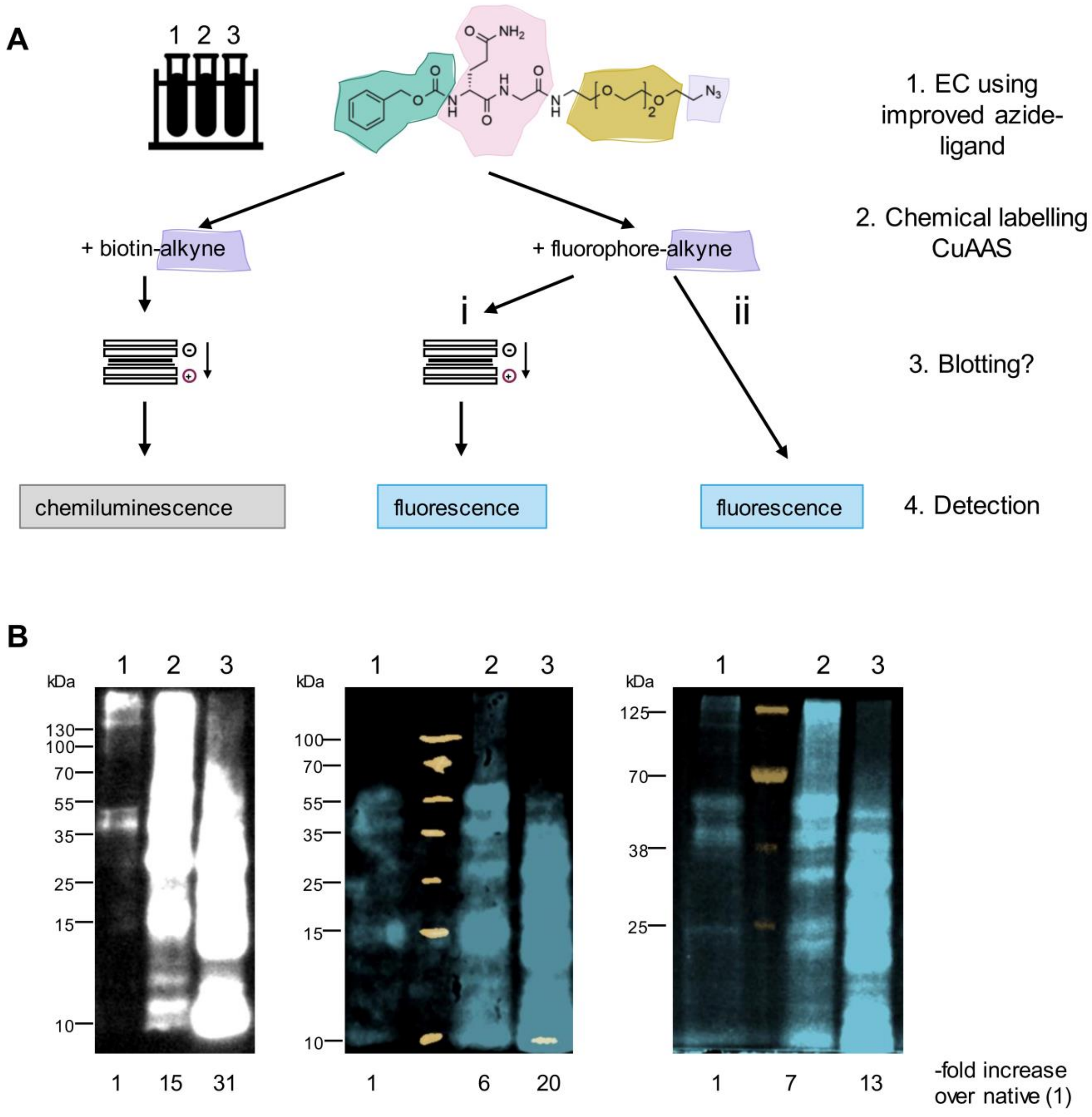

Figure S1. Evaluation of the effect of ligand composition, blotting process and detection method on the differential signal achieved in the EC. (A) Schematic of the experimental workflow. 1. EC treatment of three different Rituximab samples (1: native, 2: UV stress, 3: heat stress) using an improved mTG ligand containing an azide for CuAAS; 2. All samples divided in two and labelled via CuAAS with a biotin- and a fluorophore-alkyne; 3. All samples were resolved on SDS-PAGE, biotin-labelled samples were then blotted onto a membrane and detected via chemiluminescence, fluorescence-labelled samples were i) blotted onto a membrane and imaged at 700 nm or ii) imaged at 700 nm directly in the SDS-PAGE cassette. (B) Results of the experiment, arranged under the corresponding experimental setup from (A). Lane 1: native, lane 2: UV stress, lane 3: heat stress. White: chemiluminescence, orange: 800 nm (MW ladder), blue: 700 nm. Overall signal intensity increase over the corresponding native sample is indicated under each lane.

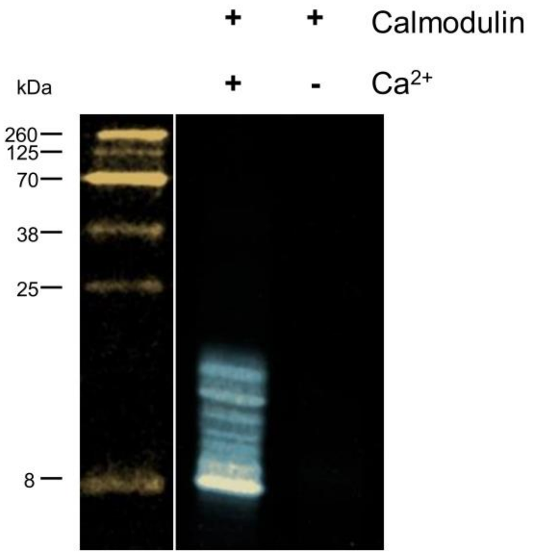

Figure S2. Overdigestion of calmodulin by 1:500 molar ratio of papain:calmodulin in the first step of a standard two-step fEC. Orange: 800 nm (MW ladder), blue: 700 nm. Depletion of calcium was achieved by addition of EDTA.

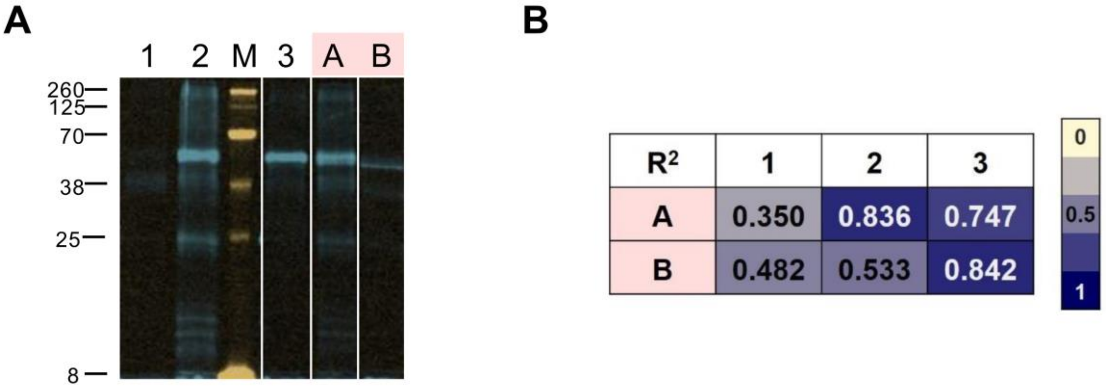

Figure S3. Classification of structural impurities in Rituximab within one PAGE after fEC. (A) Library samples (1. native, 2: 10' UV stress, 3: deglycosylated protein) and variations thereof (A: 5' UV stress, B: 20% deglycosylated protein spiked into native sample) after two-step fEC (limited proteolysis using pepsin followed by transglutamination), analysed on two SDS-PAGE runs. Orange: 800 nm (MW ladder), blue: 700 nm. (B) Heat map showing the squared correlation coefficient (R^2) determined by the described MatLab script.

References

[1] E. Dall, H. Brandstetter, *Proc Natl Acad Sci U S A* **2013**, *110*, 10940-10945.
[2] T. Loo, M. L. Patchett, G. E. Norris, J. S. Lott, *Protein Expr Purif* **2002**, *24*, 90-98.
[3] J. C. Hollerweger, I. J. Hoppe, C. Regl, L. G. Stock, C. G. Huber, U. Lohrig, H. Stutz, H. Brandstetter, *Analytical chemistry* **2018**, 5055 - 5065.
[4] S. I. Presolski, V. P. Hong, M. G. Finn, *Curr Protoc Chem Biol* **2011**, *3*, 153-162.
[5] I. Dusseldorf, Oct 18, 2019 ed., **2019**.
[6] H. Brandstetter, https://plus.ac.at/xray/repository/fluorescent-enzyme-cascade/, **2022**.

Author Contributions

I.J.H. and H.B. designed the experiments and analyzed data. I.J.H. conducted experiments. A.U. and B.P. designed and B.P. coded the Matlab prototype, U.L. and C.G.H. analyzed and discussed data. I.J.H. and H.B. wrote the manuscript. All authors read and approved the manuscript.
